# The impact of peritoneal lavage cytology in biliary tract cancer (KHBO1701): Kansai Hepato‐Biliary Oncology Group

**DOI:** 10.1002/cnr2.1323

**Published:** 2020-12-06

**Authors:** Satoshi Matsukuma, Hiroaki Nagano, Shogo Kobayashi, Hiroshi Wada, Satoru Seo, Daisuke Tsugawa, Hiroyuki Okuyama, Kenjiro Iida, Yoshiaki Ohmura, Yutaka Takeda, Atsushi Miyamoto, Shinsuke Nakashima, Terumasa Yamada, Tetsuo Ajiki, Akihito Tsuji, Kenichi Yoshimura, Hidetoshi Eguchi, Etsuro Hatano, Tatsuya Ioka, Kansai Hepato‐Biliary Oncology (KHBO) Group

**Affiliations:** ^1^ Department of Gastroenterological, Breast and Endocrine Surgery Yamaguchi University Graduate School of Medicine Yamaguchi Japan; ^2^ Kansai Hepato‐Biliary Oncology Group Osaka Japan; ^3^ Department of Gastroenterological Surgery Graduate School of Medicine, Osaka University Osaka Japan; ^4^ Department of Surgery Osaka International Cancer Institute Osaka Japan; ^5^ Department of Surgery Graduate School of Medicine, Kyoto University Kyoto Japan; ^6^ Division of Hepato‐Biliary‐Pancreatic Surgery, Department of Surgery Graduate School of Medicine, Kobe University Kobe Japan; ^7^ Department of Clinical Oncology Kagawa University Hospital Kagawa Japan; ^8^ Department of Gastroenterological Surgery Hyogo College of Medicine Nishinomiya Japan; ^9^ Department of Surgery Kansai Rosai Hospital Amagasaki Japan; ^10^ Department of Surgery Osaka National Hospital Osaka Japan; ^11^ Department of Surgery Higashiosaka City Medical Center Osaka Japan; ^12^ Medical Center for Clinical and Translational Research Hiroshima University Hospital Hiroshima Japan; ^13^ Department of Oncology Center Yamaguchi University Hospital Yamaguchi Japan

**Keywords:** cholangiocarcinoma, cytology‐positive peritoneal lavage, peritoneal metastatic recurrence

## Abstract

**Background:**

Only few studies in literature have analyzed the clinical effects of peritoneal lavage status in biliary tract cancers.

**Aim:**

We aimed to assess the effect of cytology‐positive peritoneal lavage on survival for patients with biliary tract cancer who underwent curative resection.

**Methods:**

The KHBO1701 study was a multi‐institutional retrospective study that assessed the clinical effects of peritoneal lavage cytology in biliary tract cancers. Using clinicopathological data from 11 Japanese institutions, we compared long‐term outcomes between patients with cytology‐positive and cytology‐negative peritoneal lavage.

**Results:**

Of 169 patients who underwent curative resection, 164 were cytology‐negative, and five were cytology‐positive. The incidence of portal invasion and preoperative carbohydrate antigen 19‐9 levels were higher in the cytology‐positive group than in the cytology‐negative group. The incidence of peritoneal metastatic recurrence was also higher, and overall survival tended to be worse in the cytology‐positive group. In contrast, recurrence‐free survival was similar between the cytology‐negative and cytology‐positive groups.

**Conclusions:**

The positive status of peritoneal lavage cytology could moderately affect the survival of patients with biliary tract cancers. Given that surgical resection is the only curative treatment option, it may be acceptable to resect biliary tract cancers without other non‐curative factors, regardless of peritoneal lavage cytology status.

## 1 INTRODUCTION

Biliary tract cancers (BTCs), including intrahepatic cholangiocarcinoma (ICC), extrahepatic bile duct cancer (ECC), gallbladder cancer (GBC), and ampullary region cancer (AmpCa), are intractable diseases with a dismal prognosis.^1^ Radical resection without residual tumor may be the only option for a potential cure.^2^, ^3^

The presence of cancer cells in peritoneal lavage is a predictor of subsequent peritoneal dissemination of tumors, and cytology‐positive peritoneal lavage (CY+) affects the survival of patients with gastric^4^ and pancreatic cancers.^5^ For patients with advanced gastric cancer, Japanese guidelines recommend staging laparoscopy to detect peritoneal dissemination, including CY+.^6^ A change in the cytology result from positive to negative after neoadjuvant chemotherapy has been reported to be associated with improved survival.^4^ Although CY+ is associated with poor prognosis in pancreatic cancer,^5^, ^7^, ^8^, ^9^, ^10^, ^11^ resection could improve the outcomes of patients with CY+.^11^

However, only few studies with a small number of patients have analyzed the clinical effect of CY on BTC,^12^, ^13^ and the significance of CY+ in BTC remains unknown. The major concern regarding CY+ for BTC is whether surgical resection for these tumors is justified in the absence of other non‐curative factors.

Thus, we conducted this multi‐institutional retrospective study to assess the effect of CY+ on BTC.

## 2 MATERIALS AND METHODS

We conducted this multi‐institutional retrospective study to compare the outcomes between patients with BTC having CY+ and negative CY (CY−) who underwent curative resection. The study was approved by the institutional review board of each institution (protocol number in Yamaguchi University Hospital, which was the leading institution of this study: H29‐094) and was conducted according to the ethical standards of the 2013 Declaration of Helsinki. This clinical trial has been registered at https://www.umin.ac.jp/icdr/index-j.html (identifier: UMIN000029888). Informed consent was waived because this study was a retrospective cohort study.

### 2.1 Patients and study design

Clinicopathological data of patients with BTCs who underwent curative resection from January 2013 to January 2016 were collected from 11 institutions in Japan. These data were obtained from the clinical records in each institute, and the anonymized data were sent to an independent data center—the Osaka International Cancer Institute. Clinical data included age, sex, preoperative therapy, adjuvant therapy, preoperative serum carbohydrate antigen 19‐9 **(**CA19‐9) level, tumor location, and operative procedure. Pathological data included T and N status according to the tumor‐node‐metastasis Classification of Malignant Tumours eighth edition by Union for International Cancer Control,^14^ histological type, surgical margin status, and CY. CY status was not considered as a factor of residual tumor status (R). Data management was performed at the independent data center.

The collected data were carefully analyzed, and patients who underwent non‐curative resection were excluded from this study. These included seven patients with distant metastasis, including five with paraaortic lymph node metastasis, and 30 patients who underwent microscopic non‐curative resection (R1), including patients with positive biliary margin (*n* = 22) and/or patients with cancer cells in exfoliative margin (*n* = 11). Forty‐eight patients with AmpCa were also excluded because the outcome of these patients was significantly better than that of patients with the other three types of cancer (Figures S1 and S2), and none of these patients had CY+. The clinicopathological data of patients with AmpCa are shown in Table S1.

### 2.2 Peritoneal lavage cytology

After laparotomy, the pelvic and/or subhepatic space was washed with 0.9% sodium chloride (10‐200 mL), and the peritoneal washing fluid was collected for pathological examination. Smears were prepared using centrifuged deposits, stained with Papanicolaou and/or Giemsa staining, and examined by experienced pathologists. CY+ was defined as the presence of cancer cells in peritoneal lavage. The CY status results were obtained before the resection in some centers and after resection in others. Surgical resection was performed irrespective of the CY status. Neoadjuvant and adjuvant therapies were administered to some patients according to the policy of each institute.

### 2.3 Statistical analyses

Clinicopathological and survival data were compared between the patients with CY+ and CY−. Data are presented as medians and interquartile ranges. Continuous variables were analyzed using the Mann‐Whitney *U* test, and categorical variables were analyzed using the chi‐square test or Fisher's exact test, as appropriate. The Kaplan‐Meier method was used to calculate the recurrence‐free survival (RFS) and overall survival (OS), with differences being evaluated using the log‐rank test. The cumulative incidence of peritoneal metastasis was estimated using the cumulative incidence function, taking into consideration the competing risk of death before peritoneal metastasis. The differences between the groups were compared using Gray's test.

Multivariate analysis to identify independent prognostic factors of OS was conducted using Cox proportional regression model. Several potential confounders reported as predictors for OS, including lymph node metastasis, differentiation, vascular invasion, combined vascular resection,^1^ and modified Glasgow Prognostic Score (mGPS), were included in the model.^15^

The Fine and Gray competing risks proportional hazards regression model was used to identify the independent predictors, accounting for the competing risk of death before peritoneal metastasis. Variables with *P* < .10 in the univariate analysis were included in the model for peritoneal recurrence because the predictors for peritoneal recurrence have not been fully clarified. All tests were two‐sided, and *P* < .05 was considered to be statistically significant. All statistical analyses were performed with R (The R Foundation for Statistical Computing, Vienna, Austria).

## 3 RESULTS

One hundred and eight patients with ECC, 33 patients with GBC, and 28 patients with ICC were included in this study. Among 169 patients who underwent curative resection, five patients (3.0%) had a CY+ status, and 164 patients had a CY− status. Overall, the postoperative complication rate of more than Dindo‐Clavian grade IIIa was 26.0%; however, the 90‐day mortality rate was zero.

### 3.1 Comparison of clinicopathological factors between the CY+ and CY− groups

Preoperative CA19‐9 levels in the CY+ group were significantly higher than those in the CY− group (Table 1). Fifteen patients in the CY− group and one patient in the CY+ group received preoperative therapy, including gemcitabine plus radiation,^16^ gemcitabine or S‐1 alone, gemcitabine plus S‐1 or cisplatin (GS or GC), and gemcitabine plus S‐1 plus cisplatin.^17^ Sixty‐eight patients in the CY− group and two patients in the CY+ group received postoperative adjuvant chemotherapy, including S‐1 or gemcitabine alone, and gemcitabine plus S‐1 or cisplatin. Other details of the patients in the CY+ group are shown in Table S2. None of the five patients underwent preoperative transhepatic biliary drainage, and one patient alone underwent preoperative percutaneous tumor biopsy in the CY+ group.

**TABLE 1:** Clinicopathological characteristics of patients

	Cytology‐negative (*n* = 164)	Cytology‐positive (*n* = 5)	*P*‐value
Age (years)^a^	71.0 [64‐74]	63.0 [63‐70]	.413
Sex (Female/Male)	61/103	3/2	.368
Tumor entity			1.000
ICC	27 (16.5)	1 (20.0)	
ECC	105 (64.0)	3 (60.0)	
GBC	32 (19.5)	1 (20.0)	
BMI (kg/m^2^)^a^	21.2 [19.8‐23.2]	20.5 [18.2‐23.0]	.523
mGPS 0	139 (84.8)	4 (80.0)	.712
1	8 (4.9)	0 (0.0)	
2	17 (10.4)	1 (20.0)	
Preoperative CA19‐9 (units/mL)^a^	29.1 [11.2‐149.5]	191.0 [187.5‐357.4]	.046
PET SUV max^a^ ^,^ ^b^	5.3 [3.5‐8.3]	5.4 [5.3‐10.0]	.307
Preoperative therapy	15 (9.1)	1 (20.0)	.396
Operative procedure			
Cholecystectomy including extended resection with hepatic bed	22 (13.4)	0 (0)	
Extrahepatic bile duct resection	7 (4.3)	0 (0)	
Partial hepatic resection	2 (1.2)	0 (0)	
Sectionectomy	4 (2.4)	0 (0)	
Bisectionectomy	71 (43.3)	1 (20.0)	
Trisectionectomy	6 (3.7)	1 (20.0)	
PD	47 (28.7)	3 (60.0)	
Extended hemihepatectomy + PD	5 (3.0)	0 (0)	
Combined portal vein resection	22 (13.4)	2 (40.0)	.148
Combined artery resection	11 (6.7)	1 (20.0)	.311
Differentiation			.104
Papillary adenocarcinoma	20 (12.2)	0 (0)	
Tubular adenocarcinoma			
Well‐differentiated	55 (33.5)	0 (0)	
Moderately differentiated	54 (32.9)	5 (100)	
Poorly differentiated	19 (11.6)	0 (0)	
Others	16 (9.8)	0 (0)	
ICC Tis	1	0	1.000
T1	5	0	
T2	16	1	
T3	0	0	
T4	5	0	
ECC Tis/T0	3/1^c^	0	.481
T1	13	0	
T2	41	0	
T3	39	3	
T4	8	0	
GBC Tis	1	0	.152
T1	5	0	
T2	12	0	
T3	11	0	
T4	3	1	
Serosal invasion	116 (70.7)	4 (80.0)	.606
Lymph node metastasis	55 (33.5)	2 (40.0)	1.000
Portal invasion	14 (8.5)	3 (60.0)	.007
Arterial invasion	7 (4.3)	1 (20.0)	.218
Dindo‐Clavian grade ≥ IIIa complication	42 (25.6)	2 (40.0)	.606
Postoperative hospital stay (days)^a^	37 [24‐63]	42 [31‐53]	.461
Adjuvant therapy	68 (41.5)	2 (40.0)	1.000

*Note*: Values in parentheses are percentages unless indicated otherwise.

Abbreviations: ECC, extrahepatic cholangiocarcinoma including perihilar bile duct cancer; GBC, gall bladder cancer; ICC, intrahepatic cholangiocarcinoma; mGPS, modified Glasgow prognostic score; PD, pancreaticoduodenectomy.

^a^ Values are median [Interquartile range].

^b^ SUV max in the primary site.

^c^ This case achieved complete remission after preoperative chemotherapy.

All five patients in the CY+ group had a moderately differentiated tumor. Although portal invasion was higher in the CY+ group than in the CY− group, the rate for combined portal and arterial resection was similar between the two groups.

### 3.2 Comparison of survival and recurrence pattern between the CY+ and CY− groups that underwent curative resection

**FIGURE 1:**
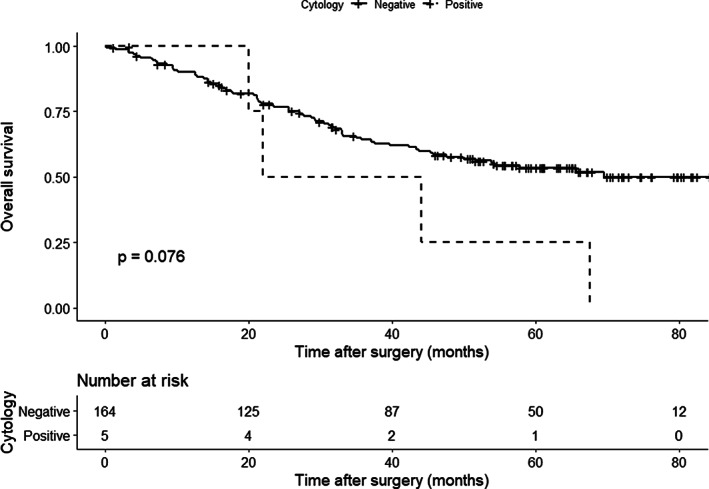
Comparison of overall survival between patients with cytology‐positive peritoneal lavage (*n* = 5, dotted black line) and cytology‐negative peritoneal lavage (*n* = 164, solid black line) who underwent curative resection

**FIGURE 2:**
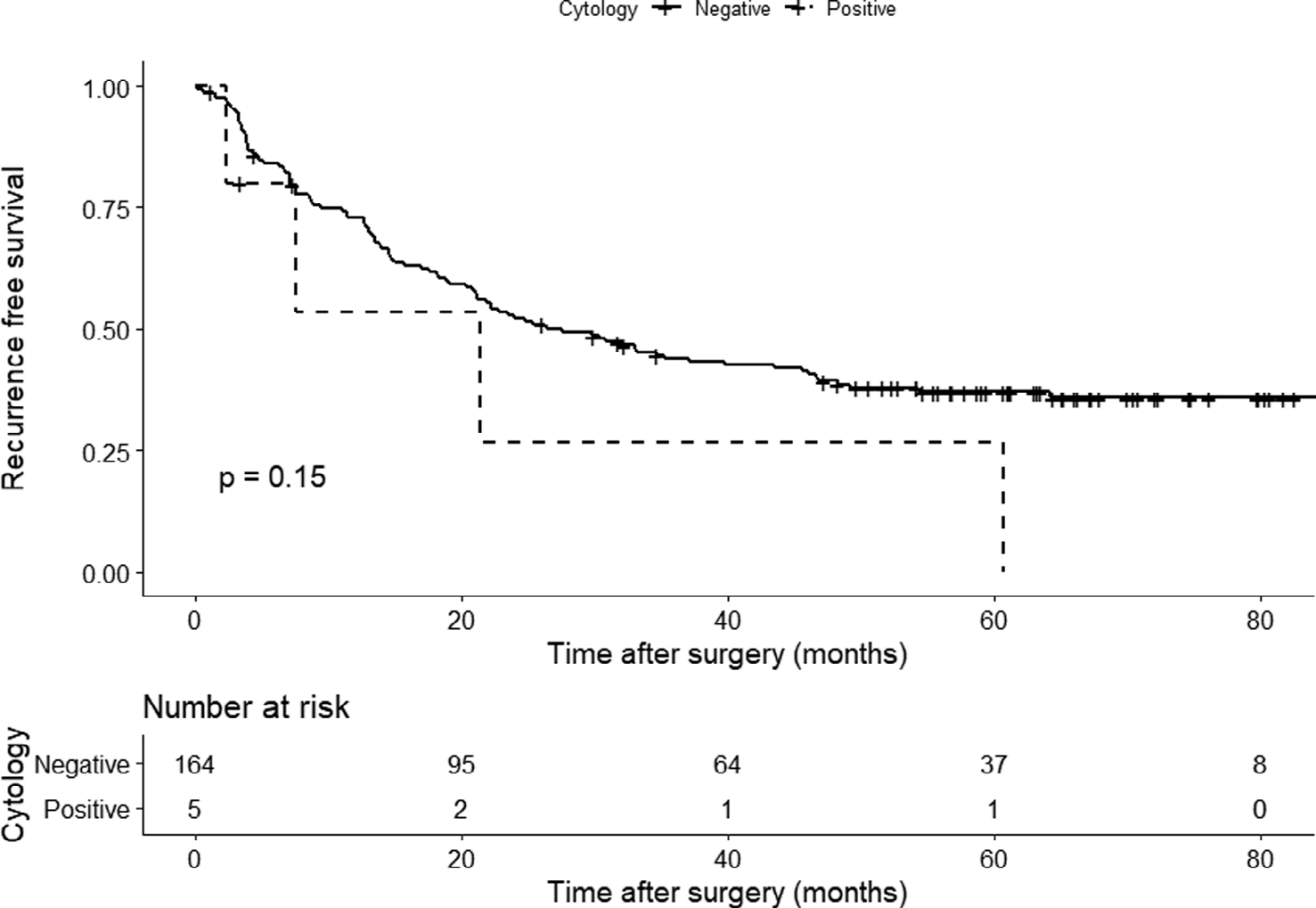
Comparison of recurrence‐free survival between patients with cytology‐positive peritoneal lavage (*n* = 5, dotted black line) and cytology‐negative peritoneal lavage (*n* = 164, solid black line) who underwent curative resection

The median duration of follow‐up was 45.3 months. Although OS tended to be worse in the CY+ group than in the CY− group (Figure 1, median survival time [MST] 33.0 vs not reached, *P* = .076), RFS was similar between the two groups (Figure 2, median 21.4 vs 26.4 months, *P* = .150). In contrast, OS and RFS in patients who did not receive preoperative therapy were similar between the CY+ (*n* = 4) and the CY− groups (*n* = 149) (MST 44.1 vs 65.7 months, *P* = .25, Figure S3 and median RFS 21.4 vs 24.0 months, *P* = .500, Figure S4, respectively).

**FIGURE 3:**
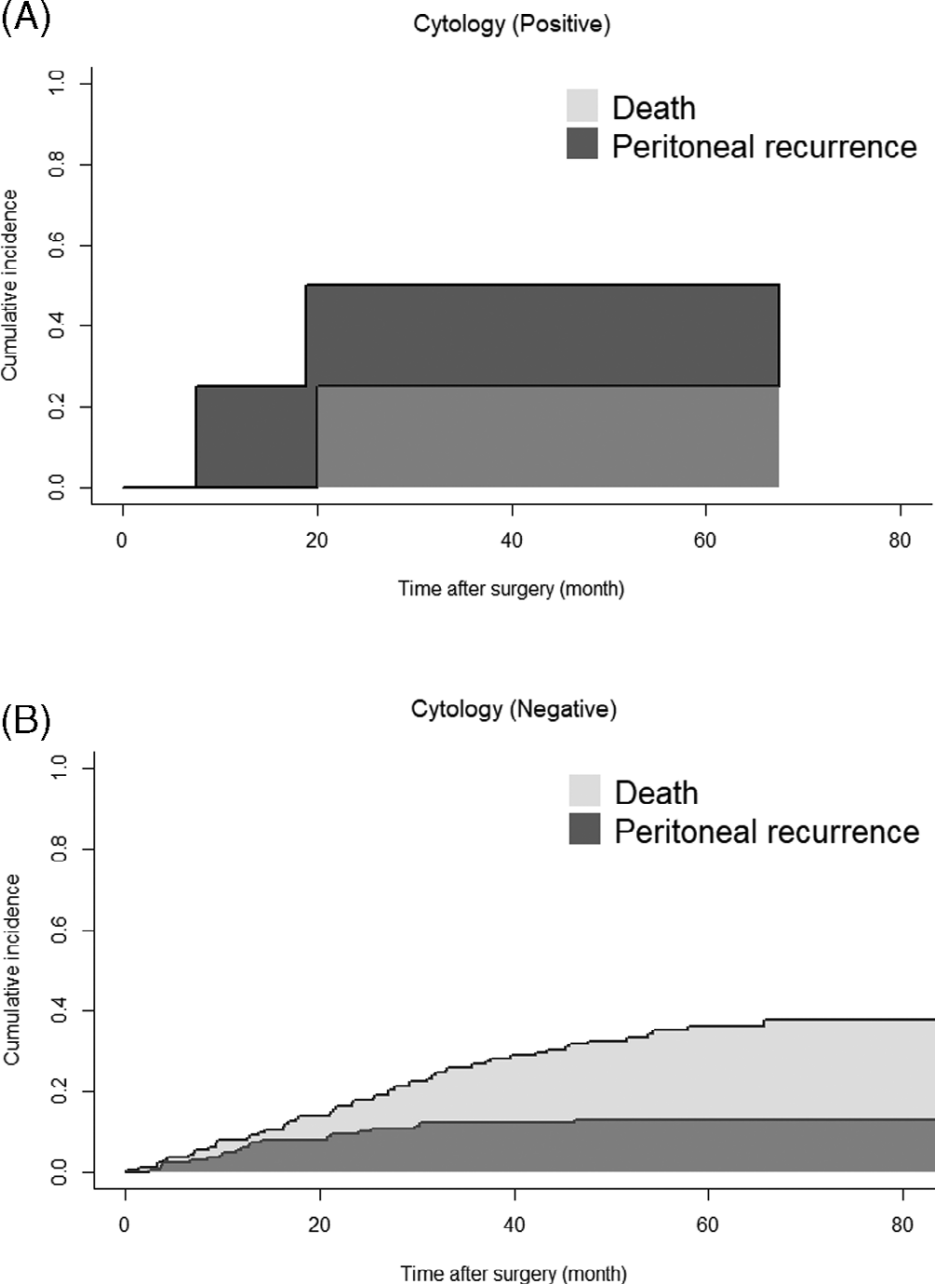
Cumulative incidence of peritoneal metastasis and death before peritoneal metastasis in patients who underwent curative resection with cytology‐positive peritoneal lavage (CY+), A, and cytology‐negative peritoneal lavage (CY−), B. The cumulative incidence of peritoneal metastasis was higher in the CY+ group than in the CY− group (*P* = .034)

**FIGURE 4:**
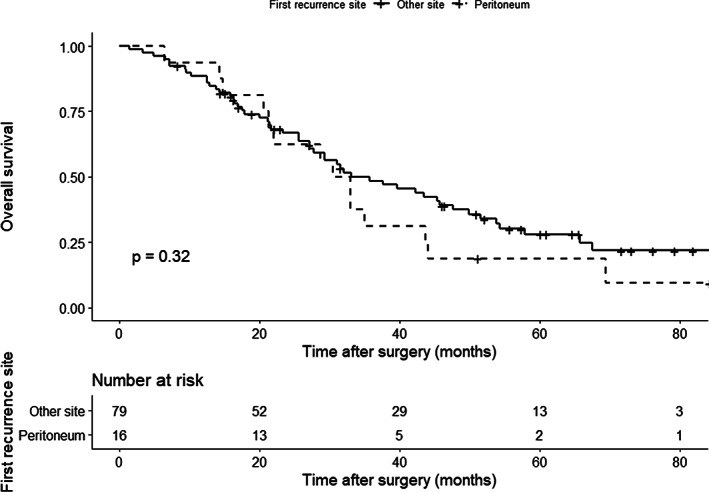
Comparison of overall survival with respect to the primary site of recurrence. Survival was similar between patients with recurrence in the peritoneum (*n* = 16, dotted black line) and in other sites (*n* = 79, solid black line). The remaining 74 patients were free of disease

**FIGURE 5:**
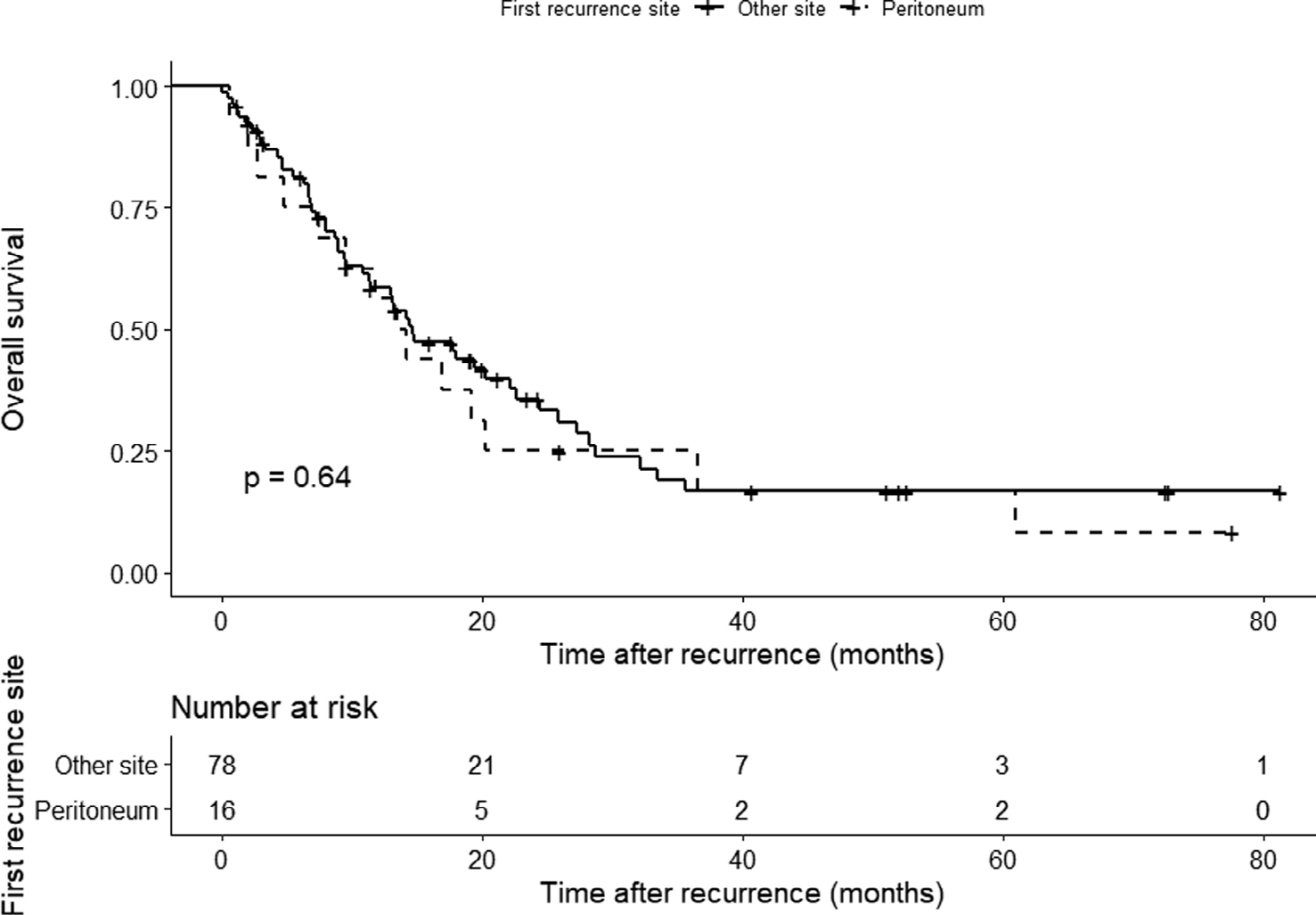
Comparison of survival after recurrence with respect to the primary site of recurrence. Survival was similar between patients with recurrence in the peritoneum (*n* = 16, dotted black line) and in other sites (*n* = 79, solid black line). The remaining 74 patients were free of disease

Overall, 95 patients experienced recurrence. The primary sites of recurrence are shown in Table 2. The proportion of peritoneal recurrence in all patients who experienced recurrence was similar between the CY+ and CY− groups (*n* = 2 [50.0%] vs *n* = 14 [15.4%], *P* = .131). The cumulative incidence of peritoneal metastasis was higher in the CY+ group than in the CY− group (Figure 3, *P* = .034), although the incidence of death prior to peritoneal metastasis was similar. However, patients with recurrence in the peritoneum (*n* = 16) had similar OS and survival after recurrence to those with recurrence in other sites (*n* = 79) (MST 31.7 vs 35.7 months, *P* = .320, Figure 4 and median survival time after recurrence 13.8 vs 14.6 months, *P* = .640, Figure 5, respectively).

**TABLE 2:** Primary site of recurrence

Recurrence (number of patients)	Cytology‐negative (*n* = 91)	Cytology‐positive (*n* = 4)
Site of first recurrence^a^		
Peritoneum	14	2
Liver	39	2
Lymph node	24	0
Local	20	0
Lung	12	0
Others	4	0

^a^ Including overlapping.

### 3.3 Prognostic factors of OS and peritoneal recurrence

The presence of lymph node metastasis, well‐differentiated tumor morphology, vascular invasion, serosal invasion, combined vascular resection, mGPS score, resection more than trisectionectomy or extended hemihepatectomy plus pancreaticoduodenectomy, and CY+ status were included in the model for OS. mGPS (score 1: hazard ratio [HR] 2.701, *P* = .043, score 2: HR 3.032, *P* < .001), well differentiation (HR 0.577, *P* = .031) and serosal invasion (HR 2.046, *P* = .030) were identified as prognostic factors of OS (Table 3).

**TABLE 3:** Univariate and multivariate analysis of prognostic factors for overall survival

	Overall survival (%)				Multivariable analysis	
	No. of patients	3 years	5 years	Univariable *P*	Hazard ratio	*P*
All patients	169	63.8	52.8	—		
Sex						
Female	64	70.8	51.7	.610		
Male	105	59.3	53.2			
Age at surgery (years)						
≥71	87	62.4	54.4	.840		
<71	82	65.4	51.3			
Intrahepatic cholangiocarcinoma						
Yes	28	64.3	46.3	.570		
No	141	63.8	54.1			
Gallbladder cancer						
Yes	33	75.0	61.1	.310		
No	136	60.9	50.7			
Extrahepatic cholangiocarcinoma						
Yes	108	60.1	51.9	.660		
No	61	70.2	54.4			
mGPS						
0	143	68.1	57.6	<.001		
1	8	50.0	37.5		2.701	.043
2	18	35.6	21.3		3.032	<.001
Preoperative therapy						
Yes	16	81.2	67.7	.200		
No	153	61.9	51.1			
Resection of ≥Hr3 or HPD						
Yes	12	40.0	15.0	.003	1.933	.084
No	157	65.7	55.4			
Combined vascular resection						
Yes	31	54.8	49.8	.350	0.864	.661
No	138	65.7	53.7			
Differentiation						
Well	75	72.1	64.1	.012	0.577	.031
Others	94	57.2	43.9			
Vascular invasion						
Yes	112	54.1	44.0	<.001	1.668	.119
No	57	83.1	69.8			
Serosal invasion						
Yes	120	53.9	45.6	<.001	2.046	.030
No	49	88.6	70.8			
Lymph node metastasis						
Yes	57	48.1	43.2	.020	1.328	.270
No	112	71.6	57.5			
Cytology						
Positive	5	50.0	25.0	.076	1.888	.240
Negative	164	64.2	53.5			
Postoperative therapy						
Yes	70	62.5	48.7	.800		
No	99	65.0	56.1			

Abbreviations: HPD, extended hemihepatectomy plus pancreaticoduodenectomy; Hr3, trisectionectomy; mGPS, modified Glasgow prognostic score.

To assess predictors for peritoneal recurrence, the presence of preoperative and postoperative therapy, vascular invasion, serosal invasion, mGPS, and CY+ were included in the model. Preoperative therapy (subdistribution HR 0.033, *P* = .002) and CY+ (subdistribution HR 4.251, *P* = .020) were identified as predictive factors of peritoneal recurrence (Table 4).

**TABLE 4:** Univariate and multivariate analysis of predictive factors for peritoneal recurrence

Variables	Univariate analysis			Multivariate analysis		
	sHR	95% CI	*P*‐value	sHR	95% CI	*P*‐value
Sex						
Female	1					
Male	1.704	0.637‐4.558	.288			
Age at surgery (years)						
≥71	1					
<71	0.566	0.225‐1.423	.226			
ICC						
No	1					
Yes	1.411	0.467‐4.183	.535			
GBC						
No	1					
Yes	0.955	0.277‐3.290	.942			
ECC						
No	1					
Yes	0.821	0.325‐2.076	.677			
mGPS						
0	1					
1	3.627	0.817‐16.100	.090	3.993	0.805‐19.808	.090
2	2.257	0.644‐7.912	.203	1.226	0.253‐5.949	.800
Preoperative therapy						
No	1					
Yes	0.060	0.009‐0.427	.005	0.033	0.004‐0.281	.002
Resection of ≥Hr3 or HPD						
No	1					
Yes	1.488	0.359‐6.178	.584			
Combined vascular resection						
No	1					
Yes	0.906	0.292‐2.811	.864			
Differentiation						
Well	1					
Others	0.767	0.305‐1.927	.572			
Vascular invasion						
No	1					
Yes	6.757	1.203‐37.959	.030	4.254	0.394‐45.909	.233
Serosal invasion						
No	1					
Yes	5.212	1.159‐23.430	.031	3.872	0.525‐28.557	.184
Lymph node metastasis						
No	1					
Yes	1.434	0.580‐3.545	.435			
Cytology						
Negative	1					
Positive	4.452	1.005‐19.719	.049	4.251	1.255‐14.398	.020
Postoperative therapy						
No	1					
Yes	2.757	1.069‐7.114	.036	2.331	0.770‐7.061	.134

Abbreviations: ECC, extrahepatic cholangiocarcinoma; GBC, gallbladder cancer; HPD, extended hemihepatectomy plus pancreaticoduodenectomy; Hr3, trisectionectomy; ICC, intrahepatic cholangiocarcinoma; mGPS, modified Glasgow prognostic score; sHR, subdistribution hazard ratio.

## 4 DISCUSSION

In this study, we demonstrated that the survival of patients with BTC who underwent curative resection with CY+ tended to be worse than that of those with CY−. These results are partially inconsistent with those of previous studies^12^, ^13^ that found that the CY status did not affect patients' outcomes. This discrepancy may be due to the small number of cases in previous studies, and the accumulation of more CY+ cases than in our study could further clarify the difference in prognosis. In addition, our results showed that the cumulative incidence of peritoneal metastasis after surgery was higher in the CY+ group than in the CY− group.

The major concern regarding CY+ BTC is whether surgical resection for these tumors is justified in the absence of other non‐curative factors. Our results of a median RFS of 21.4 months and a median OS of 33 months in CY+ cases, and the fact that the survival of patients who underwent combination therapy with GC for unresectable lesions was approximately 11 months^18^, ^19^ implied that resection for BTC with CY+ might be justified in cases without other non‐curative factors in the present situation where effective preoperative and postoperative adjuvant therapy is not established.

The accuracy of preoperative imaging modalities, including computed tomography, magnetic resonance imaging, and positron emission tomography, in detecting locally advanced tumors, liver metastasis, and lymph node metastasis has improved dramatically in the last decade^20^, ^21^, ^22^; however, it is difficult to detect small peritoneal metastatic nodules using these modalities. In this situation, staging laparotomy or laparoscopy is effective to determine the presence of radiologically occult metastasis of BTC.^23^ Moreover, CY status could predict peritoneal metastatic recurrence and could be a complement to staging laparotomy or laparoscopy.

The effect of CY status on the OS of patients with BTC is too small compared with that of patients with gastric^24^ and pancreatic cancers.^5^, ^7^, ^8^, ^9^, ^10^ The main reason for this weak effect on OS could arise from other strong prognostic factors of BTC, including lymph node metastasis^25^, ^26^, ^27^ and vascular invasion.^28^, ^29^ However, both of these strong prognostic factors were not significant, and mGPS, serosal invasion, and not well‐differentiated types were prognostic factors in this study. The cause of the weak effect is unknown; however, our study included several cancer types, which may have led to this result.

We could no suggest the reason as to why the number of patients with CY+ in BTC was much smaller in this study (3.0%) than in previous reports (7.7%^12^ and 9.8%^13^). Although it is difficult to draw a definitive conclusion because of small number of CY+ and peritoneal metastatic recurrence in CY+ cases, our results implied a higher cumulative incidence of peritoneal metastasis in patients with CY+ after curative resection. Additionally, some studies showed that higher seeding metastasis occurs after percutaneous transhepatic biliary drainage and resection than after endoscopic biliary drainage for perihilar cholangiocarcinoma^30^ and distal cholangiocarcinoma,^31^ which suggests that cancer cells that have invaded the abdominal cavity are considered to settle in the peritoneum at a relatively high rate. In the present study, one alone of the five patients in the CY+ group underwent percutaneous tumor biopsy. Therefore, the correlation between preoperative procedure and CY+ was unclear.

The development of effective preoperative and/or postoperative chemotherapy is essential for improving the outcome of patients with CY+ BTC. GC^18^, ^19^ or GS,^32^ or triplet chemotherapy with gemcitabine, cisplatin, and S‐1^17^ are promising preoperative chemotherapy, and postoperative therapy with capecitabine^33^ or S‐1^34^ is also promising. Moreover, hyperthermic intraperitoneal chemotherapy could be a choice for patients with CY+ at risk of developing peritoneal metastasis.^35^

This study has some limitations. First, this was a retrospective study that included some bias in indication and method of peritoneal lavage cytology. Thus, further prospective studies defining indications and methods of peritoneal lavage cytology to assess the incidence and effect of CY+ on survival are needed. Second, the status of cytology was diagnosed in each institute; therefore, unexpected bias could have occurred. Third, the small number of CY+ cases in this study was a limitation. However, the increase in peritoneal recurrence in patients with CY+ in our study could indicate the usefulness of peritoneal lavage cytology in BTC.

In conclusion, the positive status of peritoneal lavage cytology could moderately affect the survival of patients with BTC with increasing the incidence of peritoneal recurrence. Considering that surgical resection is the only potentially curative therapeutic option, it may be acceptable to resect BTCs without other non‐curative factors, regardless of the CY status.

## AUTHOR CONTRIBUTIONS

**Satoshi Matsukuma:** Data curation; formal analysis; investigation; writing‐original draft; writing‐review and editing. **Hiroaki Nagano:** Conceptualization; investigation; methodology; project administration; supervision; writing‐original draft; writing‐review and editing. **Shogo Kobayashi:** Data curation; investigation; methodology. **Hiroshi Wada:** Data curation; investigation; methodology. **Satoru Seo:** Data curation; investigation; methodology. **Daisuke Tsugawa:** Data curation; investigation; methodology. **Hiroyuki Okuyama:** Data curation; investigation; methodology. **Kenjiro Iida:** Data curation; investigation; methodology. **Yoshiaki Ohmura:** Data curation; investigation; methodology. **Yutaka Takeda:** Data curation; formal analysis; investigation. **Atushi Miyamoto:** Data curation; investigation; methodology. **Shinsuke Nakashima:** Data curation; investigation; methodology. **Terumasa Yamada:** Data curation; investigation; methodology. **Tetsuo Ajiki:** Data curation; investigation; methodology; supervision. **Akihito Tsuji:** Data curation; investigation; methodology. **Ken‐ichi Yoshimura:** Data curation; investigation; methodology. **Hidetoshi Eguchi:** Data curation; investigation; supervision. **Etsuro Hatano:** Conceptualization; project administration; supervision. **Tatsuya Ioka:** Conceptualization; investigation; project administration; supervision.

## CONFLICT OF INTEREST

The authors declare no conflicts of interest.

## ETHICAL STATEMENT

The study was approved by the Institutional Review Board of each institution (protocol number in Yamaguchi University Hospital, which was the leading institution of this study: H29‐094) and was conducted according to the ethical standards of the 2013 Declaration of Helsinki. This clinical trial has been registered at https://www.umin.ac.jp/icdr/index-j.html (identifier: UMIN000029888). Informed consent was waived because this study was a retrospective cohort study.

## Supporting information

**Figure S1**. Overall survival according to the disease. The survival of patients with ampullary region cancer (solid purple line) was significantly better than that of patients with other three cancers (*P* = 0.04).
Abbreviations ECC, extrahepatic cholangiocarcinoma including perihilar bile duct cancer; GBC, gall bladder cancer; ICC, Intrahepatic cholangiocarcinoma.

**Figure S2**. Recurrence‐free survival according to the disease. The survival of patients with ampullary region cancer (solid purple line) was significantly better than that of patients with other three cancers (*P* = 0.0046).
Abbreviations ECC, extrahepatic cholangiocarcinoma including perihilar bile duct cancer; GBC, gall bladder cancer; ICC, Intrahepatic cholangiocarcinoma.

**Figure S3**. Comparison of overall survival between patients with cytology‐positive peritoneal lavage (*n* = 4, solid blue line) and cytology‐negative peritoneal lavage (*n* = 149, solid red line) who underwent curative resection without preoperative therapy.

**Figure S4**. Comparison of recurrence‐free survival between patients with cytology‐positive peritoneal lavage (*n* = 4, solid blue line) and cytology‐negative peritoneal lavage (*n* = 149, solid red line) who underwent curative resection without preoperative therapy.

**Table S1**. Clinicopathological characteristics of patients with ampullary region cancer.

**Table S2**. Supporting Information.

## Data Availability

The data that support the findings of this study are available from the corresponding author upon reasonable request.
