# Supplementary figures and images for: The impact of peritoneal lavage cytology in biliary tract cancer (KHBO1701): Kansai Hepato‐Biliary Oncology Group
Source: Cancer Rep (Hoboken). 2020 Dec 6;4(2):e1323. doi: 10.1002/cnr2.1323 (PMC8451372; doi:10.1002/cnr2.1323)

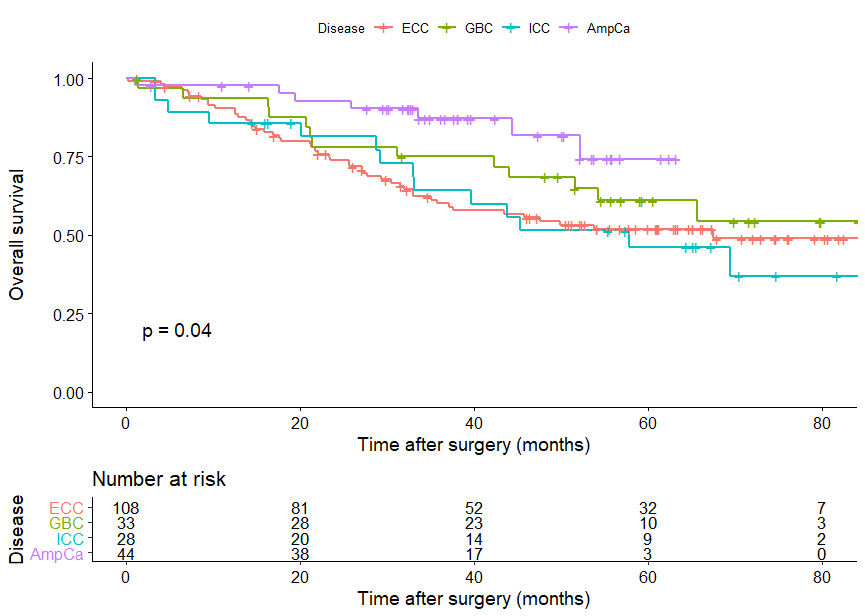

Supplement: Supplementary file 1 — Figure S1. Overall survival according to the disease. The survival of patients with ampullary region cancer (solid purple line) was significantly better than that of patients with other three cancers (P = 0.04). Abbreviations ECC, extrahepatic cholangiocarcinoma including perihilar bile duct cancer; GBC, gall bladder cancer; ICC, Intrahepatic cholangiocarcinoma. [file CNR2-4-e1323-s004.tiff]

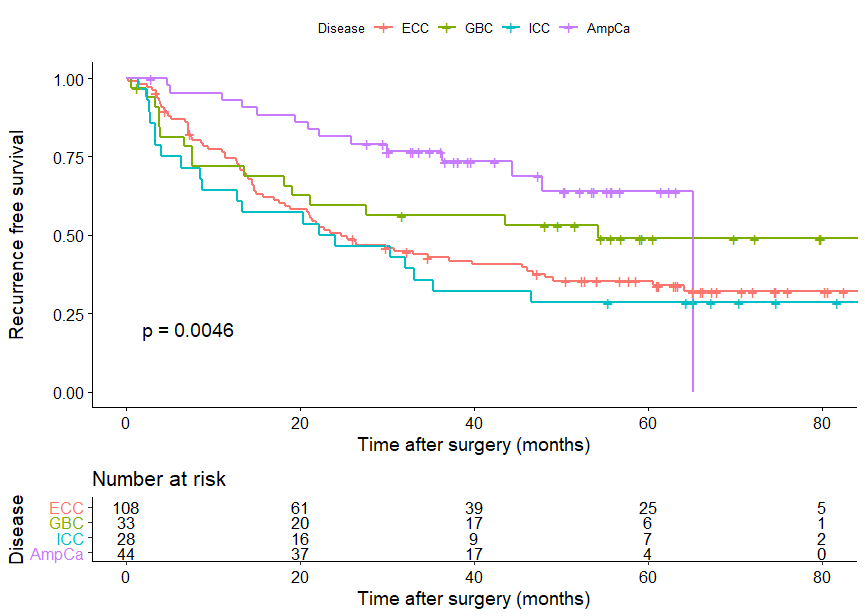

Supplement: Supplementary file 2 — Figure S2. Recurrence‐free survival according to the disease. The survival of patients with ampullary region cancer (solid purple line) was significantly better than that of patients with other three cancers (P = 0.0046). Abbreviations ECC, extrahepatic cholangiocarcinoma including perihilar bile duct cancer; GBC, gall bladder cancer; ICC, Intrahepatic cholangiocarcinoma. [file CNR2-4-e1323-s001.tiff]

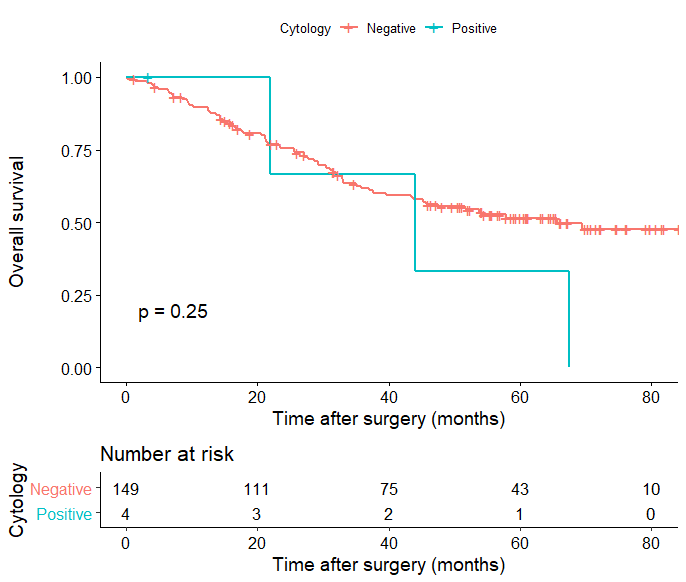

Supplement: Supplementary file 3 — Figure S3. Comparison of overall survival between patients with cytology‐positive peritoneal lavage (n = 4, solid blue line) and cytology‐negative peritoneal lavage (n = 149, solid red line) who underwent curative resection without preoperative therapy. [file CNR2-4-e1323-s003.tiff]

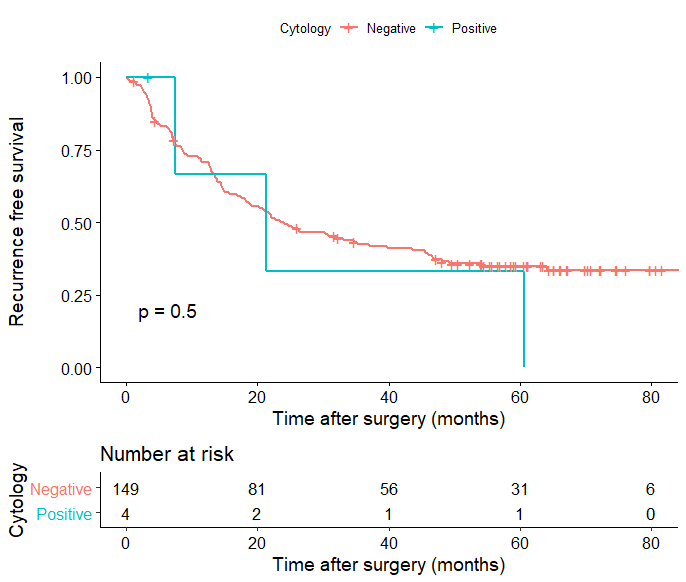

Supplement: Supplementary file 4 — Figure S4. Comparison of recurrence‐free survival between patients with cytology‐positive peritoneal lavage (n = 4, solid blue line) and cytology‐negative peritoneal lavage (n = 149, solid red line) who underwent curative resection without preoperative therapy. [file CNR2-4-e1323-s006.tiff]
