# Supplementary material for: The impact of peritoneal lavage cytology in biliary tract cancer (KHBO1701): Kansai Hepato‐Biliary Oncology Group
Source: Cancer Rep (Hoboken). 2020 Dec 6;4(2):e1323. doi: 10.1002/cnr2.1323 (PMC8451372; doi:10.1002/cnr2.1323)
Supplement: Supplementary file 5 — Table S1. Clinicopathological characteristics of patients with ampullary region cancer. [file CNR2-4-e1323-s005.docx]

Supplementary Table 1 Clinicopathological characteristics of patients with ampullary region cancer

Variable

N 44

Age (years) † 74.0 [66.5 – 76.5]

Sex (Female/Male) 23 : 21

BMI (kg/m^2^) † 22.1 [18.9 - 24.3]

Preoperative CA19-9 (units/ml) † 14.0 [8.0 – 32.0]

Operative procedure

PD 43 (97.7)
 TP 1 (2.3)

Cytology positive 0

Differentiation ‡

Papillary adenocarcinoma 1 (2.3)

Tubular adenocarcinoma

Well differentiated 32 (72.7)

Moderately differentiated 8 (18.2)

Poorly differentiated adenocarcinoma 2 (4.5)

Others 1 (2.1)

T stage ‡

Tis 4 (9.1)

T1 19 (43.2)

T2 9 (20.5)

T3 10 (22.7)

T4 2 (4.5)

Lymph node metastasis 13 (29.5)

Stage ‡

0 4 (9.1)

I 24 (54.5)

II 13 (29.5)

III 2 (4.5)

IV 1 (2.3)

Postoperative hospital stays (days) † 33.0 [23.5 – 54.5]

90-day mortality 0

Preoperative therapy 1 (2.3)

Postoperative therapy 10 (22.7)

Site of first recurrence
 Liver 5

Lymph node 1

Lung 1

Remnant pancreas 1

Peritoneum 1

Values in parentheses are percentages unless indicated otherwise; † values are median [Interquartile range], ‡ Classification according to the Classification of biliary tract cancers established by Japanese Society of Hepato-Biliary-Pancreatic Surgery: 3^rd^ English edition

PD, pancreaticoduodenectomy; TP, total pancreatectomy
