# Supplementary material for: The impact of peritoneal lavage cytology in biliary tract cancer (KHBO1701): Kansai Hepato‐Biliary Oncology Group
Source: Cancer Rep (Hoboken). 2020 Dec 6;4(2):e1323. doi: 10.1002/cnr2.1323 (PMC8451372; doi:10.1002/cnr2.1323)
Supplement: Supplementary file 6 — Table S2. Supporting Information. [file CNR2-4-e1323-s002.docx]

Supplementary Table 2

Case Entity procedure PBD Biospy pT Neoadjuvant Adjuvant Site of first recurrence RFS † Status OS †

1 ECC PD ENBD none T3 none none Liver 60.6 Dead 67.5

2 ECC PD ENBD none T3 none none none 3.4 Alive 3.4

3 ECC PD ERBD Endoscopic T3 none Gemcitabine Peritoneum 21.4 Dead 22.0

4 GBC Right lobectomy ENBD Endoscopic T4 none Gemcitabine Peritoneum 7.5 Dead 44.1

5 ICC Trisectionectomy none Percutaneous T2 GC none Liver 2.4 Dead 20.0

PBD, preoperative biliary drainage; RFS, recurrence free survival; OS, overall survival; ECC, extrahepatic cholangiocarcinoma including perihilar bile duct cancer; GBC, gall bladder cancer; ICC, intrahepatic cholangiocarcinoma; PD, pancreaticoduodenectomy; ENBD, endoscopic naso-biliary drainage; ERBD, endoscopic retrograde biliary drainage; GC, gemcitabine plus cisplatin

† months
